# Different patterns of *pfcrt* and *pfmdr1* polymorphism in *Plasmodium falciparum* isolates from Tehama region, Yemen

**DOI:** 10.7717/peerj.2191

**Published:** 2016-07-12

**Authors:** Wahib M. Atroosh, Hesham M. Al-Mekhlafi, Adel Al-Jasari, Hany Sady, Salwa S. Dawaki, Fatin N. Elyana, Mona A. Al-Areeqi, Nabil A. Nasr, Awatif M. Abdulsalam, Lahvanya R. Subramaniam, Meram Azzani, Init Ithoi, Yee Ling Lau, Johari Surin

**Affiliations:** 1 Department of Parasitology, Faculty of Medicine, University of Malaya, Kuala Lumpur, Malaysia; 2 Unit of Microbiology and Parasitology, Department of Para-Clinic, Faculty of Medicine and Health Sciences, University of Aden, Khormaksar, Aden, Yemen; 3 Endemic and Tropical Diseases Unit, Medical Research Centre, Jazan University, Jazan, Kingdom of Saudi Arabia; 4 Department of Parasitology, Faculty of Medicine and Health Sciences, Sana’a University, Sana’a, Yemen; 5 National Malaria Control Programme, Ministry of Health and Population, Sana’a, Yemen; 6 Department of Social and Preventive Medicine, Faculty of Medicine, University of Malaya, Kuala Lumpur, Malaysia; 7 Centre for Research and Innovation, Taylor’s University, Subang Jaya, Selangor, Malaysia

**Keywords:** Malaria, *Plasmodium falciparum*, Yemen, Drug resistance, *pfcrt*, *pfmdr1*, Chloroquine

## Abstract

**Introduction.** Despite the efforts of the malaria control programme, malaria morbidity is still a common health problem in Yemen, with 60% of the population at risk. *Plasmodium falciparum* is responsible for 99% of malaria cases. The emergence in Yemen of parasite resistance to chloroquine (CQ) prompted the adoption of artemisinin combination therapy (ACT) in 2009, which involves the use of artesunate plus sulphadoxine-pyrimethamine (AS + SP). However, CQ was retained as the drug of choice for vivax malaria. To assess the impact of the change in the malaria treatment policy five years after its introduction, the present study investigated the mutations in the CQ resistance transporter (*pfcrt*) and multidrug resistance 1 (*pfmdr1*) genes.

**Method.** A molecular investigation of 10 codons of *pfcrt* (72–76, 220, 271, 326, 356, and 371) and five codons of *pfmdr1* (86, 184, 1034, 1042, and 1246) was conducted on *P. falciparum* isolates from districts with the highest malaria endemicity in the Hodeidah and Al-Mahwit governorates in Tehama region, Yemen. A total of 86 positive cases of falciparum monoinfection were investigated for the presence of mutations related to CQ and other antimalarials using a PCR-RFLP assay.

**Results.** There was a wide prevalence of *pfcrt* gene mutations with the *pfcrt* 76T CQ resistance marker being predominant (97.7%). The prevalence of other *pfcrt* mutations varied from high (75E: 88%) to moderate (74I: 79.1%, 220S: 69.8%, 271E and 371I: 53.5%) or low (326S: 36%, 72S: 10.5%). Mutated *pfcrt* 72–76 amino acids haplotypes were highly prevalent (98.8%). Among these, the CVIET classic, old-world African/Southeast Asian haplotype was the most predominant, and was mostly found in the isolates from the Khamis Bani Saad district of Al-Mahwit (93.1%) and the AdDahi district of Hodeidah (88.9%). However, it was only found in 26.3% of the isolates from the Bajil district of Hodeidah. Surprisingly, the SVMNT new-world South American haplotype was exclusively detected in 9.3% of the isolates from the Bajil district of Hodeidah. Mutations at Y184F of *pfmdr1* were found in all isolates (100%) from all districts. The mutation for codons 1034C and 86Y were found only in the isolates from the AdDahi and Khamis Bani Saad districts. Overall, the AdDahi and Khamis Bani Saad districts were similar in terms of carrying most of the mutations in the *pfcrt* and *pfmdr1* genes, while there was a lower prevalence of mutation in the isolates from the Bajil district.

**Conclusion.** The high prevalence of mutations in *pfcrt* 5 years after the official cessation of CQ use against *P. falciparum* suggests that there is sustained CQ pressure on *P. falciparum* isolates in the study area. Moreover, the low prevalence of mutations in the *pfmdr1* gene could be a good indicator of the high susceptibility of *P. falciparum* isolates to antimalarials other than CQ. A new strategy to ensure the complete nationwide withdrawal of CQ from the private drug market is recommended.

## Introduction

Chloroquine (CQ), a safe and cheap antimalarial, has been the drug of choice for treating uncomplicated falciparum malaria over the past few decades. However, its effectiveness has been greatly hampered by the emergence of resistant *Plasmodium falciparum* strains that emerged along the Thai–Cambodian border early in 1957 and then spread to other foci in Asia, and also to South America, Papua New Guinea, and Africa (Moore & Lanier, 1961; Harinasuta, Suntharasamai & Viravan, 1965; Grimmond, Donovan & Riley, 1976; Campbell et al., 1979; Fogh, Jepsen & Effersoe, 1979). The emergence and spread of *P. falciparum* strains that are resistant to CQ and other antimalarials has necessitated a switch to new drugs to ensure maximum effectiveness and prevent the development of ongoing parasite resistance. It has been suggested that a combination therapy rather than monotherapies is highly efficient for falciparum malaria case management and malaria control.

Currently, artemisinin combination therapy (ACT) is the cornerstone of malaria treatment policies and control worldwide (Nosten & White, 2007; White, 2008; World Health Organization, 2015). Interestingly, the replacement of CQ with an ACT in areas where falciparum isolates were CQ-resistant resulted in the re-emergence of CQ-sensitive strains a few years after the cessation of CQ use (Liu et al., 1995; Kublin et al., 2002; Kublin et al., 2003; Mita et al., 2004).

Chloroquine resistance is determined by the major point mutation at codon 76 of the *P. falciparum* CQ resistance transporter (*pfcrt*) gene, through the substitution of lysine amino acid (K) by threonine (T) (Fidock et al., 2000), which is highly correlated with increased clinical CQ tolerance and treatment failure (Djimde et al., 2001; Wellems & Plowe, 2001; Wongsrichanalai et al., 2002; White, 2004). However, the accumulation of mutations along the *pfcrt* gene, especially at codons A220S, Q271E, N326S, I356T, and R371, in addition to the leader *pfcrt* K76T point mutation was found significantly associated with CQ resistance (Djimde et al., 2001). Also, point mutations in the *P. falciparum* multidrug resistance 1 (*pfmdr1*) gene have been reported to modulate sensitivity and resistance to multiple antimalarials (Basco et al., 1995; Reed et al., 2000; Sisowath et al., 2005).

In Yemen, malaria is still a major public health problem that threatens more than 60% of the population, with *P. falciparum* causing 99% of the malaria cases (World Health Organization, 2015). The emergence in Yemen of parasite resistance to CQ as well as to other antimalarials prompted the adoption of ACT in 2009, which includes artesunate plus sulphadoxine-pyrimethamine (*AS* + *SP*) and artemether plus lumefantrine (AL) as the first line and second line treatment, respectively, for uncomplicated falciparum malaria infections. However, CQ was retained as the drug of choice for vivax malaria.

Some *in vivo* and *in vitro* studies have been conducted in different malaria-endemic districts of Yemen in order to evaluate the efficacy of CQ against *P. falciparum* isolates. These studies found that CQ resistance varied from 16.1% in the Taiz governorate (Alkadi, Al-Maktari & Nooman, 2006) to more than 40% in the Lahj (Mubjer et al., 2011) and Hodeidah governorates (Al-Maktari et al., 2003; Al-Shamahy et al., 2007). However, despite these reports and the introduction of a new malaria treatment policy, CQ continues to be available in drug markets and private drug stores in Yemen, is still prescribed for falciparum malaria in some private health facilities, and is also used by individuals for self-administration, especially in remote malarious areas (Bashrahil, Bingouth & Baruzaig, 2010; Ghouth, 2013).

Some molecular studies have reported a high prevalence of *pfcrt* 76T mutations (ranging from 88% to 100%) in different districts of Yemen (Al-Mekhlafi et al., 2011; Mubjer et al., 2011; Abdul-Ghani, Farag & Allam, 2013; Al-Hamidhi et al., 2013). Nevertheless, there is still a paucity of information about the full genetic profile of *pfcrt* and *pfmdr1* in the country. Yet, such genetic markers are useful tools for monitoring parasite susceptibility to various antimalarials. In addition, they help researchers to monitor changes in parasite genotypes after a change in treatment policy. Therefore, the present study aimed to investigate the susceptibility of *P. falciparum* to CQ (and some other antimalarials) five years after the adoption of an ACT by conducting a molecular analysis of the related mutations in the *pfcrt* and *pfmdr1* genes in isolates from the Tehama region in northwestern Yemen. Furthermore, because there is a diverse market for antimalarials in this region, improving our knowledge about drug pressure would be useful in targeting interventions aimed at reducing inappropriate drug use in the region.

**Figure 1:**
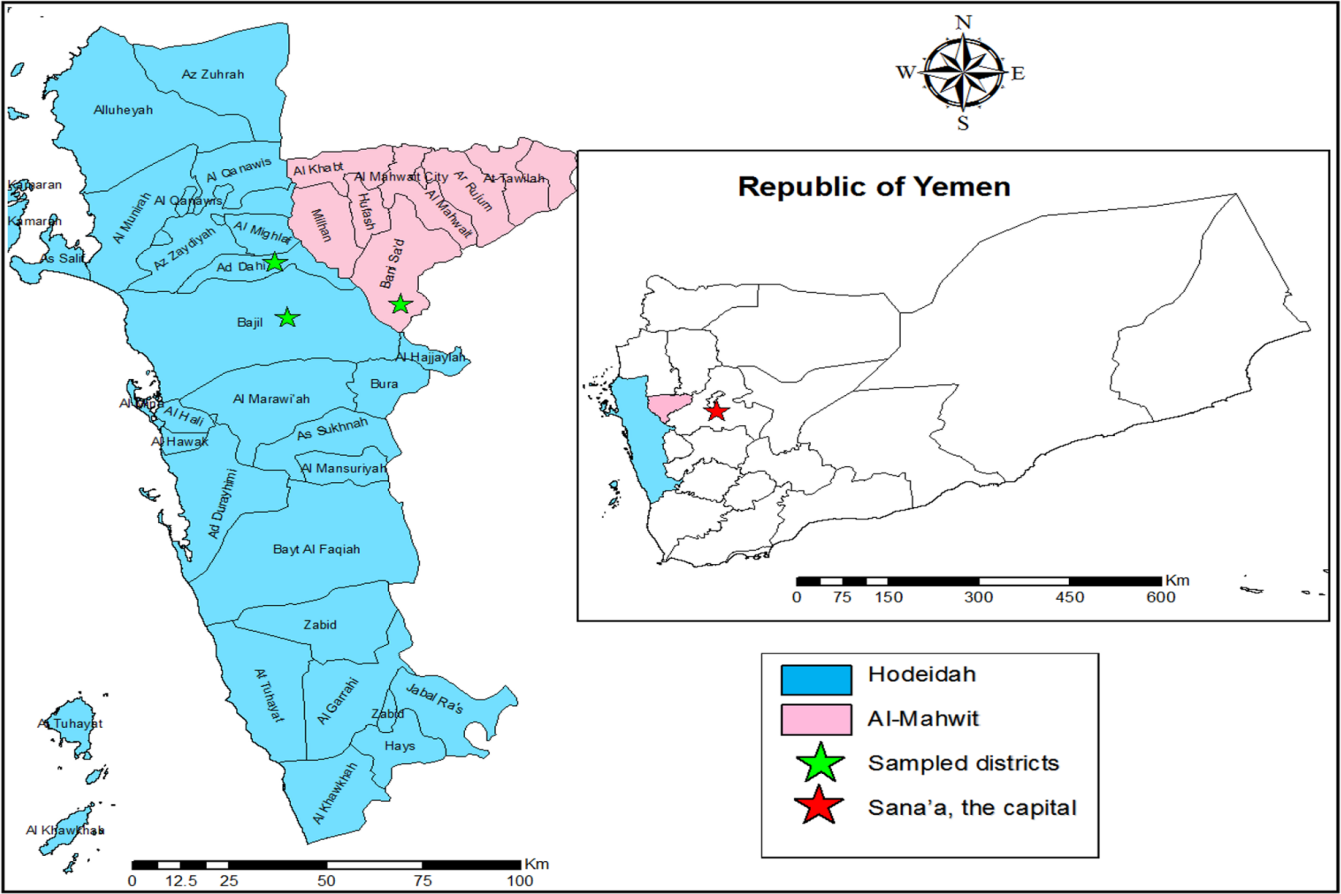
A geographic map showing study area (Hodeidah and Al-Mahwit governorates).

## Materials and Methods

### Study area and subjects

An active case survey that targeted individuals with fever (as a group suspected to have malaria) was conducted from March to May 2014 in two malaria-endemic districts of the Hodeidah governorate and one in the Al-Mahwit governorate in the Tehama region in northwestern Yemen (Fig. 1). The Hodeidah governorate (14.79°N, 42.97°E) is located about 226 km from Sana’a, the capital of Yemen. Hodeidah is a coastal area along the Red Sea with a total area of 117,145 km^2^ and a total population of 2.16 million (National Information Centre, Yemen, 2014). The Al-Mahwit governorate (16.25°N, 44.717°E) is located between Hodeidah and Sana’a (about 111 km west of Sana’a), covers a total area of 2,858 km^2^, and has a total population of 597,000 (National Information Centre, Yemen, 2014). The climate in the Tehama region is a combination of tropical monsoons with occasional rain in summer and dry weather in winter with a mean rainfall of 200 mm/year. The mean temperature is 37.5 °C in summer and 24 °C in winter with humidity ranging between 70% and 90%. Malaria transmission is perennial, but there is a high malaria transmission peak from January to March. Transmission is known to be more prevalent in Hodeidah.

Villages in the districts with the highest malaria endemicity in the region were chosen based on the national malaria records for the period 2010–2013 that were provided by the National Malaria Control Programme (NMCP), Yemen. Based on these records, samples were collected from 10 villages in the AdDahi and Bajil districts in Hodeidah and the Khamis Bani Saad district in Al-Mahwit. The villages in question were Halalah, Al-Huamarah, Al-Meshaahra, Al-Rakib, Al-Rufae, Al-Sharjah, Deer Shareef, Kidf Zumailah, Shat Hajal, and Siraj. Samples were also collected from the city of Bajil.

The present study employed an *in vivo* efficacy trial that was designed to investigate the effectiveness of the *AS* + *SP* therapy in treating uncomplicated malaria and that used the World Health Organization (WHO) 28-day follow-up protocol. Initially, 622 individuals with fever in the 11 mentioned sites were examined. From this number, 86 individuals with falciparum malaria monoinfection, who voluntarily agreed to take part in the *in vivo* study, were recruited based on the inclusion criteria in the WHO guidelines (WHO, 2009). All 86 were considered for the investigation of different molecular markers related to resistance to antimalarial drugs, including the *pfcrt* and *pfmdr1* genes.

### *Plasmodium falciparum* isolates

A finger-prick blood sample was collected from each participant for use in the RDT test (*CareStart*™ Malaria HRP2-RDT) and for preparing thick and thin blood film and a filter paper blood spot. Blood films were stained with 5% of buffer-diluted Giemsa stain for 30 min and examined microscopically for malaria parasites. Filter papers were left to dry in air away from dust and then kept in an aluminium pouch together with desiccated silicon bags until used.

Parasite species and stages were recorded and parasitaemia (parasite density) was determined by counting only the asexual stages against 300 white blood cells (WBCs) and then multiplying by 25, assuming the average of the total WBC count of the individuals was equal to 7,500 cells per µl of blood (Billo et al., 2013). The level of parasitaemia was graded as low (<1,000 parasites/µl of blood), moderate (1,000–9,999 parasites/µl of blood), or severe (≥10,000 parasites/µl of blood).

### DNA extraction

Two to three discs (6 mm diameter) of 3MM Whatman’s filter paper blood spots (cut by a flamed-sterile puncher) were used for DNA extraction, which was performed using a Qiagen blood and tissue kit (Cat. no. 69506; QIAGEN, DNeasy^®^ Blood & Tissue Kit, Germany) according to the manufacturer’s instructions. The DNA was eluted using 100 µl of AE (10 mM Tris-Cl; 0.5 mM EDTA; pH 9.0) elution buffer (included in the kit) and kept at –20 °C until used.

### Detection of gene mutations in *pfcrt* and *pfmdr1*

All extracted DNA samples of *P. falciparum* were subjected to mutation analysis using polymerase chain reaction (PCR) amplification followed by restricted fragment length polymorphism (RFLP) to investigate the mutations in CQ resistance transporter (*pfcrt*) and multidrug resistance1 (*pfmdr1*) genes.

Amplification of *pfcrt* was performed using the protocol designed by Djimde et al. (2001) with the aim of detecting mutations at codons 72–76, 220, 271, 326, 356, and 371 of the *pfcrt* gene and at codons 86 and 1246 of the *pfmdr1* gene. The other codon mutations of **pfmdr1** (184, 1034, and 1042) were analysed using the PCR-RFLP protocol in Duraisingh et al. (2000). However, the PCR amplification and restriction enzyme (RE) digestion in the protocol was modified for codon 184 by designing a forward primer MDR184-F (5′-ATAATAATCCTGGATCTAAATTAAGA-3′) to replace A4 to amplify an amplicon of 155 bp instead of 560 bp and by using Swa1 endonuclease which cuts once into 123 bp and 32 bp for the mutated allele but not the wild.

Restriction enzyme digestion was done in 20 µl of reaction mixture that consisted of 6–8 µl of PCR product, 1X of the specific buffer (included in the RE kit), and 1 unit of the specific RE (New England Biolabs Inc., Hitchin, UK), then was incubated for 15–60 min (based on the RE) according to the manufacturer’s instructions, and then visualised in 2.5%–4.0% of TAE buffered agarose gel (based on the size of the cleaves) stained with Sybr^®^ safe DNA gel stain (Invitrogen, USA) using a Bio-Rad Molecular Imager Gel Doc XR System (Bio-Rad, Hercules, CA, USA).

Genomic DNA of *P. falciparum* strains 3D7 (MRA-102G) and HB3 (MRA-155G) were used as positive controls for the wild types of *pfcrt* and *pfmdr1*, while the Dd2 strain (MRA-150G) was used as positive control for the mutated types. All the strains were provided by the Malaria Research and Reference Reagents Resources Center (MR4, ATCCW, Manassas VA, USA).

The study protocol was approved by the Medical Ethics Committee of the University of Malaya Medical Centre, Kuala Lumpur (Ref. 974.19), and by the Ministry of Health and Population, in conjunction with the National Malaria Control Programme in Yemen.

## Results

A total of 86 malaria-positive individuals participated in the present study; 40 (46.5%) were male and 46 (53.5%) were female. The ages of participants ranged from 8 months to 65 years, with a mean age of 12.4 years. The age group of 5–15 years represented 58.1% of the study participants. Asexual parasite density (parasitaemia) varied from 561 to 55,555 parasites/µl, with a mean parasitaemia of 8,199 parasites/µl.

The *pfcrt* gene mutations were screened as molecular markers of CQ, and possibly of other antimalarials such as amodiaquine and lumefantrine. All isolates were successfully amplified (100%). Mutation at *pfcrt* 76T was found at a virtual fixation level in almost all districts (97.7%), followed by 88.4% for position 75E. The prevalence of mutations at other codons of *pfcrt* was found to vary from moderate as in codons 74I (79.1%), 220S (69.8%), and 271E and 371I (53.5%) to low as in 326S (36%) and 72S (10.5%). None of the isolates considered in the present study carried the mutated type at codon 356 of *pfcrt* (see Table 1).

Eight haplotypes of *pfcrt* 72–76 amino acids were found to be present in the study area. The CVIET classical, old-world African/Southeast Asian haplotype was the most prevalent among all *pfcrt* haplotypes (77.9%), followed by SVMNT (9.3%) and CVMET (7%). Only one isolate (1.2%) was found to carry each of the other mutated haplotypes, namely CVMEK, CVINT, SVMET and CVMNT, as well as the CVMNK wild haplotype.

Among all 10 types of codon mutation in *pfcrt*, sextuple mutations (i.e., mutations at six codons) had the highest presence among the isolates (33.7%), followed by quadruple mutations (four codons) with 23.3%, quintuple (five codons) with 14.4%, septuple (seven codons) with 12.2%, and triple (three codons) with 8.1%. The frequency of double and single mutations was very low; 3.5% and 1.2%, respectively.

As regards the results of the mutation analysis of the *pfmdr1* gene, *P. falciparum* field isolates from the Tehama region showed at least a single point mutation in the *pfmdr1* gene (see Table 2). Mutation at position 184 of *pfmdr1* was found at the fixation level (100%), together with a low prevalence of mutation for codons 1034 and 86 of *pfmdr1* of 20.9% and 16.3%, respectively. No mutation was detected for codons 1042 and 1246.

**Table 1:** Frequency distribution of *pfcrt* mutations and haplotypes for *P. falciparum* isolates from different districts of Tehama, Yemen.

Marker		AdDahi		Bajil		Khamis Bani Saad		Total	
		*n*	%	*n*	%	*n*	%	*n*	%
Crt	wild	9	100	10	52.6	58	100	77	89.5
72	mutated	0	0	9	47.4	0	0	9	10.5
Crt	wild	1	11.1	14	73.7	3	5.2	18	20.9
74	mutated	8	88.9	5	26.3	55	94.8	68	79.1
Crt	wild	0	0	8	42.1	2	3.4	10	11.6
75	mutated	9	100	11	57.9	56	96.6	76	88.4
Crt	wild	0	0	0	0	2	3.4	2	2.3
76	mutated	9	100	19	100	56	96.6	84	97.7
Crt	wild	4	44.4	4	21.1	18	31	26	30.2
220	mutated	5	55.6	15	78.9	40	69	60	69.8
Crt	wild	0	0	18	94.7	8	13.8	26	30.2
271	mutated	9	100	1	5.3	50	86.2	60	69.8
Crt	wild	5	55.6	2	10.5	48	82.8	55	64
326	mutated	4	44.4	17	89.5	10	17.2	31	36
Crt	wild	3	33.3	18	94.7	19	32.8	40	46.5
371	mutated	6	66.7	1	5.3	39	67.2	46	53.5
**Haplotypes**	CV**IET**	8	88.9	5	26.3	54	93.1	67	77.9
	**S**VMN**T**	0	0	8	42.1	0	0	8	9.3
	CVM**ET**	1	11.1	5	26.3	0	0	6	7
	CV**I**N**T**	0	0	0	0	1	1.2	1	1.2
	CVM**E**K	0	0	0	0	1	1.2	1	1.2
	CVMNK	0	0	0	0	1	1.2	1	1.2
	CVMN**T**	0	0	0	0	1	1.2	1	1.2
	**S**VM**ET**	0	0	1	1.2	0	0	1	1.2

**Table 2:** Frequency distribution of *pfmdr1* mutations and haplotypes for *P. falciparum* isolates from different districts of Tehama, Yemen.

Marker		AdDahi		Bajil		Khamis Bani Saad		Total	
		*n*	%	*n*	%	*n*	%	*n*	%
Mdr1	wild	5	55.6	19	100	48	82.8	72	83.7
86	mutated	4	44.4	0	0	10	17.2	14	16.3
Mdr1	wild	8	88.9	19	100	41	70.7	68	79.1
1034	mutated	1	11.1	0	0	17	29.3	18	20.9
**Haplotypes**	N**F**SND	4	44.4	19	100	32	55.2	55	64
	N**FC**ND	1	11.1	0	0	16	27.6	17	19.8
	**YF**SND	4	44.4	0	0	9	15.5	13	15.1
	**YF****C**ND	0	0	0	0	1	1.7	1	1.2

The NFSND single-mutated haplotype of phenyl alanine amino acid at position 184 was predominant (64%), followed by NFCND (19.8%), and YFSND (15.1%), with an overall presence of 34.9% for all double-mutated haplotypes. The YFCND triple-mutated haplotype was found in only one isolate (1.2%) carrying the mutated amino acids tyrosine (codon 86), phenyl alanine (184), and cysteine (1034).

Interestingly, falciparum malaria in the study area, which consisted of districts known to have the highest malaria endemicity in the country, showed unexpected variation in *pfcrt* and *pfmdr1* gene mutations. Isolates from the AdDahi district in Hodeidah and the Khamis Bani Saad district in Al-Mahwit were almost similar in terms of carrying the majority of mutated alleles for most of the codons, and consequently carrying the mutated haplotypes. In contrast, the malaria isolates from the Bajil district in Hodeidah were found to be mostly of the wild type and consequently carried less mutated haplotypes.

Isolates from the study area showed a high prevalence of CQ resistant haplotypes, with a predominance of CVIET classical old-world African/Southeast Asian haplotypes in the Khamis Bani Saad district of Al-Mahwit (93.1%) and the AdDahi district in Hodeidah (88.9%) compared to only 26.3% for isolates from the Bajil district of Hodeidah. In the same context, mutation at the *pfcrt* 72 codon was only reported in isolates from the Bajil district (47.4%), whereas it was totally absent from the other two districts. Accordingly, the SVMNT haplotype was found exclusively in 42.1% of isolates from Bajil. (Refer to Table 1.)

Similarly, mutations at *pfmdr1* showed a geographic variation in terms of *pfmdr1* allele and haplotype frequencies. That is to say, in addition to the fixation level of mutation at *pfmdr1* 184, mutations at 86 and 1034 of *pfmdr1* were found only in the AdDahi and Khamis Bani Saad districts, whereas they were not present in isolates from the Bajil district. The triple-mutated haplotype of 86, 1084 and 1034 (YFCND) was found in one isolate from Khamis Bani Saad. (See Table 2.)

## Discussion

The present study provides information on the *pfcrt* and *pfmdr1* genetic profile of *P. falciparum* isolates from the districts with the highest malaria endemicity in the Tehama region of Yemen. The isolates were found to mostly carry mutated alleles, especially for *pfcrt* codons 74I, 75E, 76T, 220S, 271E, and 371I, while no mutations were detected at codon 356 of *pfcrt*. This is consistent with a previous study conducted in Yemen that reported a high prevalence of mutated alleles for codons 74I and 75E (89%) and 76T (93%) (Al-Hamidhi et al., 2013). The present study found that the overall prevalence of *pfcrt* 76T was 74%–100%, which is similar to the figure previously reported for isolates from other parts of Yemen (Al-Mekhlafi et al., 2011; Mubjer et al., 2011; Bamaga, Mahdy & Lim, 2015).

The high level of mutation at position 76 of *pfcrt* (96.6%–100%) appears to indicate that, 5 years on from changing the malaria treatment policy to one that is ACT based and the official cessation of CQ use for *P. falciparum*, the re-emergence of a CQ-sensitive strain of *P. falciparum* has not yet occurred in the study area. One possible explanation for the virtual fixation of the *pfcrt* 76 mutation in the study area could be the continued availability of CQ; it is still being prescribed to falciparum malaria patients in Yemen particularly in private health facilities and is also being used as a self-administered treatment (Bashrahil, Bingouth & Baruzaig, 2010; Ghouth, 2013; Bamaga et al., 2014). Moreover, CQ has not yet been totally withdrawn from governmental drug stores as it is still the drug of choice for treating vivax malaria infections.

The findings of the present study support those of previous research conducted elsewhere in Yemen that have reported 79%–100% mutated alleles for *pfcrt* 76T (Al-Mekhlafi et al., 2011; Mubjer et al., 2011; Abdul-Ghani, Farag & Allam, 2013; Al-Hamidhi et al., 2013). The finding in previous studies such as these that there was a decrease in the prevalence of the *pfcrt* 76T mutated allele and a re-emergence of CQ-sensitive strains some years after abandoning CQ was controversial, particularly as many studies in Africa documented an increase in the re-emergence of the wild type of *pfcrt* 76 after complete CQ withdrawal (Laufer et al., 2006; Mohammed et al., 2013; Mbogo et al., 2014; Mekonnen et al., 2014). In contrast, South American isolates of *P. falciparum* have been reported to retain their mutated types despite the cessation of CQ use (Vieira et al., 2004; Griffing et al., 2010; Adhin, Labadie-Bracho & Bretas, 2013). Similarly, studies conducted in Ethiopia have found a fixation of *pfcrt* 76T mutations after cessation of CQ use for more than a decade (Mula et al., 2011; Golassa et al., 2014).

The results of the present study also revealed that the CVIET classical old-world, African/ Southeast Asian mutated haplotype was predominant (77.9%) among all *pfcrt* haplotypes in the study area and that seven other haplotypes were also present, including 9.3% (eight cases) of the SVMNT new-world South American mutated haplotype (only in the Bajil district) and 7.0% (six cases) of CVMET. This finding is in agreement with the only two other available studies based in Yemen that attempted to examine the mutations along the *pfcrt* gene (Al-Hamidhi et al., 2013; Mubjer et al., 2011). For instance, CVIET was reported for the majority of the isolates from different parts of Yemen (Dhamar 100%, Taiz 88%, and Hodeidah 70.6%), with a presence of only 4% of the SVMNT haplotype in isolates from the Hodeidah governorate (Al-Hamidhi et al., 2013). Similarly, CVIET was the only haplotype reported for isolates from the Al-Musaimeer malaria-endemic district in Lahj Province (Mubjer et al., 2011).

Interestingly, the present study found the SVMNT haplotype exclusively in 42.1% of isolates from the Bajil district in Hodeidah. This finding might be due to a ‘parasite response’ to a particular drug pressure other than CQ. However, data on the use of a unique antimalarial in this particular area were not available, so it is not possible to relate the occurrence of the SVMNT haplotype to the increased use of any of the antimalarials. A previous study conducted in Tanzania reported the unusual existence of the SVMNT haplotype in Africa and attributed the finding, without supporting evidence, to the increased drug pressure of amodiaquine (AQ) use in the area under study (Alifrangis et al., 2006).

Moreover, malaria transmission in the Bajil district, an area with the SVMNT haplotype, was found to be lower than that in the AdDahi and Khamis Bani Saad districts. This is in line with the study on Tanzania, which reported a higher existence of the SVMNT mutated haplotype in areas with low malaria transmission than in areas with high transmission (Alifrangis et al., 2006). As mentioned above, it has been suggested that the SVMNT haplotype occurs as a response to the intense pressure of AQ, and *P. falciparum* isolates with SVMNT are supposedly less susceptible to AQ monotherapy and combination therapy. Interestingly, the NMCP in Yemen conducted six *in vivo* clinical trials for CQ and AQ drug efficacy from 2002 to 2004; four studies looked at CQ efficacy and found that there was 30%–57% treatment failure, while two studies monitored the efficacy of AQ monotherapy and artesunate-amodiaquine (AST-AQ) combination therapy and found 44.3% and 18.5% treatment failure rate, respectively (National Malaria Control Programme (NMCP), 2013, unpublished data).

The existence of SVMNT in Bajil rather than in the other two districts raises an interesting question about the source of the evolution of this CQ resistant haplotype in the country. Typically, SVMNT is associated with new-world South American or Papua New Guinea isolates, and importation of this haplotype from South America or Papua New Guinea, or even from the Philippines or India in Asia, was logically unaccepted based on the population characteristics of the study area. Hence, the apparent independent evolution of SVMNT in Bajil isolates will need further study to confirm the finding presented herein. However, the results of a study conducted in Papua New Guinea suggest that there is a recombination of de novo point mutation that transforms the old-world classical African CVIET haplotype into a new-world South American SVMNT one (Mehlotra et al., 2001).

As regards the *pfmdr1* gene, the present study found that mutations varied among the isolates from the three districts, from fixation (100%) for 184F to 20.9% for 1034C and 16.3% for 86Y, while no mutation was detected for the 1042 and 1246 codons. These results are consistent with those of a previous study in Hodeidah that reported a high prevalence (99%) of 184F and a relatively low existence (20%) of the 86Y mutated allele (Al-Hamidhi et al., 2013). Likewise, a recent study conducted in Hadramout in the southeastern part of Yemen, which has low malaria transmission, reported a low frequency (16.7%) of *pfmdr1* 86 mutated allele (Bamaga, Mahdy & Lim, 2015). However, a high prevalence (70%) for mutations of 1034C and 1042D has been reported for the Taiz, Dhamar, and Hodeidah governorates (Al-Hamidhi et al., 2013).

It has been found that polymorphism in the *pfmdr1* gene is related to an increase in parasite tolerance/resistance to some antimalarials (Gamo, 2014). Nevertheless, the findings regarding the association of mutations in the *pfmdr1* codons with antimalarial drug resistance are mixed. For instance, 86Y has been linked to CQ resistance, while mutations at positions 184, 1034, 1042, and 1246 were found to be mostly related to resistance to AQ, mefloquine, halofantrine, and quinine (Reed et al., 2000; Danquah et al., 2010; Gamo, 2014). Moreover, the occurrence of *pfmdr1* N86 and 184F has been found to increase with the use of lumefantrine (Sisowath et al., 2007; Nwakanma et al., 2008; Malmberg et al., 2013). In the same vein, another study reported an increase of *pfmdr1* N86 and 184F, together with the wild alleles of D1246, and concluded that this was linked to the extensive and prolonged use of AQ in combination with artesunate (Fröberg et al., 2012). In other studies, *P. falciparum* isolates carrying *pfmdr1* haplotypes of mutated 86Y and Y184 wild alleles were found to be associated with the usage of CQ or AQ, which continued to change over time with changes in the antimalarials used. For instance, mutated 86Y and Y184 wild alleles changed to a haplotype carrying wild N86 and 184F mutated alleles years after the implementation of ACT, and the selection of parasites to one of artemisinin’s derivatives (Humphreys et al., 2007; Dlamini, Beshir & Sutherl, 2010; Mungthin et al., 2010; Thomsen et al., 2011). Overall, the findings of the present study provide a broad view on the polymorphism of *pfcrt* and *pfmdr1* genes in areas with the highest level of malarial transmission in Yemen, 5 years after official cessation of CQ.

## Conclusion

The present study aimed to provide a genetic profile of *pfcrt* and *pfmdr1* as molecular markers of the *P. falciparum* parasite’s resistance to antimalarials, especially CQ. The high prevalence of *pfcrt* mutations and mutated haplotypes suggests a high CQ resistance in *P. falciparum* and a continuation of CQ pressure in the study area. It is therefore recommended that the availability of CQ in private hospitals, clinics, and drugstores should be controlled to ensure its complete withdrawal. Moreover, treatment of vivax malaria using CQ should only be available in governmental health facilities to prevent the random, uncontrolled use of CQ.

## Additional Information and Declarations

### Data Availability

The following information was supplied regarding data availability: All data related to this work are included in the manuscript.

## References

[ref-1] Abdul-Ghani R, Farag HF, Allam AF (2013). Sulfadoxine-pyrimethamine resistance in *Plasmodium falciparum*: a zoomed image at the molecular level within a geographic context. ACTA Tropica.

[ref-2] Adhin MR, Labadie-Bracho M, Bretas G (2013). Molecular surveillance as monitoring tool for drug-resistant *Plasmodium falciparum* in Suriname. American Journal of Tropical Medicine and Hygiene.

[ref-3] Al-Hamidhi S, Mahdy MA, Al-Hashami Z, Al-Farsi H, Al-Mekhlafi AM, Idris MA (2013). Genetic diversity of *Plasmodium falciparum* and distribution of drug resistance haplotypes in Yemen. Malaria Journal.

[ref-4] Al-Maktari MT, Bassiouny HK, Al-Hamd ZS, Assabri AM, El-Massry AG, Shatat HZ (2003). Malaria status in Al-Hodeidah Governorate, Yemen: malariometric parasitic survey & chloroquine resistance *P. falciparum* local strain. Journal of the Egyptian Society of Parasitology.

[ref-5] Al-Mekhlafi AM, Mahdy MA, Al-Mekhlafi HM, Azazy AA, Fong MY (2011). High frequency of *Plasmodium falciparum* chloroquine resistance marker (Pfcrt T76 mutation) in Yemen: an urgent need to re-examine malaria drug policy. Parasites & Vectors.

[ref-6] Al-Shamahy H, Al-Harazy A, Harmal N, Al-Kabsi A (2007). The prevalence and degree of resistance of *Plasmodium falciparum* to first-line antimalarial drugs: an *in vitro* study from a malaria endemic region in Yemen. Annals of Saudi Medicine.

[ref-7] Alifrangis M, Dalgaard MB, Lusingu JP, Vestergaard LS, Staalsoe T, Jensen ATR, Enevold A, Rønn AM, Khalil IF, Warhurst DC, Lemnge MM, Theander TG, Bygbjerg IC (2006). Occurrence of the Southeast Asian/South American SVMNT haplotype of the chloroquine-resistance transporter gene in *Plasmodium falciparum* in Tanzania. Journal of Infectious Diseases.

[ref-8] Alkadi H, Al-Maktari M, Nooman M (2006). Chloroquine-resistant *Plasmodium falciparum* local strain in Taiz Governorate, Republic of Yemen. Chemotherapy.

[ref-9] Bamaga O, Mahdy M, Lim Y (2015). Survey of chloroquine-resistant mutations in the *Plasmodium falciparum* pfcrt and pfmdr-1 genes in Hadhramout, Yemen. ACTA Tropica.

[ref-10] Bamaga O, Mahdy M, Mahmud R, Lim Y (2014). Malaria in Hadhramout, a southeast governorate of Yemen: prevalence, risk factors, knowledge, attitude and practices (KAPs). Parasites & Vectors.

[ref-11] Basco LK, Le Bras J, Rhoades Z, Wilson CM (1995). Analysis of pfmdr1 and drug susceptibility in fresh isolates of *Plasmodium falciparum* from Subsaharan Africa. Molecular and Biochemical Parasitology.

[ref-12] Bashrahil KA, Bingouth AS, Baruzaig AS (2010). Antimalarial drugs: availability and mode of prescribing in Mukalla, Yemen. Eastern Mediterranean Health Journal.

[ref-13] Billo MA, Diakité M, Dolo A, Diallo M, Poudiougou B, Diawara SI, Johnson ES, Rice JC, Krogstad DJ, Doumbo OK (2013). Inter-observer agreement according to malaria parasite density. Malaria Journal.

[ref-14] Campbell CC, Chin W, Collins WE, Teutsch SM, Moss DM (1979). Chloroquine-resistant *Plasmodium falciparum* from East Africa: cultivation and drug sensitivity of the Tanzanian I/CDC strain from an American tourist. Lancet.

[ref-15] Danquah I, Coulibaly B, Meissner P, Petruschke I, Müller O, Mockenhaupt FP (2010). Selection of pfmdr1 and pfcrt alleles in amodiaquine treatment failure in north-western Burkina Faso. ACTA Tropica.

[ref-16] Djimde A, Doumbo OK, Cortese JF, Kayentao K, Doumbo S, Diourté Y, Coulibaly D, Dicko A, Su XZ, Nomura T, Fidock DA, Wellems TE, Plowe CV (2001). A molecular marker for chloroquine-resistant falciparum malaria. The New England Journal of Medicine.

[ref-17] Dlamini SV, Beshir K, Sutherl CJ (2010). Markers of anti-malarial drug resistance in *Plasmodium falciparum* isolates from Swaziland: identification of pfmdr1-86F in natural parasite isolates. Malaria Journal.

[ref-18] Duraisingh MT, Jones P, Sambou I, Von Seidlein L, Pinder M, Warhurst DC (2000). The tyrosine-86 allele of the pfmdr1 gene of *Plasmodium falciparum* is associated with increased sensitivity to the anti-malarials mefloquine and artemisinin. Molecular and Biochemical Parasitology.

[ref-19] Fidock DA, Nomura T, Talley AK, Cooper RA, Dzekunov SM, Ferdig MT, Ursos LM, Sidhu AB, Naudé B, Deitsch KW, Su XZ, Wootton JC, Roepe PD, Wellems TE (2000). Mutations in the *P. falciparum* digestive vacuole transmembrane protein PfCRT and evidence for their role in chloroquine resistance. Molecular Cell.

[ref-20] Fogh S, Jepsen S, Effersoe P (1979). Chloroquine-resistant *Plasmodium falciparum* malaria in Kenya. Transactions of the Royal Society of Tropical Medicine and Hygiene.

[ref-21] Fröberg G, Jörnhagen L, Morris U, Shakely D, Msellem MI, Gil JP, Björkman A, Mårtensson A (2012). Decreased prevalence of *Plasmodium falciparum* resistance markers to amodiaquine despite its wide scale use as ACT partner drug in Zanzibar. Malaria Journal.

[ref-22] Gamo F-J (2014). Antimalarial drug resistance: new treatments options for Plasmodium. Drug Discovery Today. Technologies.

[ref-23] Ghouth ASB (2013). Availability and prescription practice of anti-malaria drugs in the private health sector in Yemen. Journal of Infection in Developing Countries.

[ref-24] Golassa L, Enweji N, Erko B, Aseffa A, Swedberg G (2014). High prevalence of pfcrt-CVIET haplotype in isolates from asymptomatic and symptomatic patients in south-central Oromia, Ethiopia. Malaria Journal.

[ref-25] Griffing S, Syphard L, Sridaran S, McCollum AM, Mixson Hayden T, Vinayak S, Villegas L, Barnwell JW, Escalante AA, Udhayakumar V (2010). Pfmdr1 amplification fixation of pfcrt chloroquine resistance alleles in *Plasmodium falciparum* in Venezuela. Antimicrobial Agents and Chemotherapy.

[ref-26] Grimmond TR, Donovan KO, Riley ID (1976). Chloroquine resistant malaria in Papua New Guinea. Papua New Guinea. Papua New Guinea Medical Journal.

[ref-27] Harinasuta T, Suntharasamai P, Viravan C (1965). Chloroquine-resistant falciparum malaria in Thailand. Lancet.

[ref-28] Humphreys GS, Merinopoulos I, Ahmed J, Whitty CJ, Mutabingwa TK, Sutherland CJ, Hallett RL (2007). Amodiaquine and artemether-lumefantrine select distinct alleles of the *Plasmodium falciparum* mdr1 gene in Tanzanian children treated for uncomplicated malaria. Antimicrobial Agents and Chemotherapy.

[ref-29] Kublin JG, Cortese JF, Njunju EM, Mukadam RA, Wirima JJ, Kazembe PN, Djimdé AA, Kouriba B, Taylor TE, Plowe CV (2003). Reemergence of chloroquine-sensitive *Plasmodium falciparum* malaria after cessation of chloroquine use in Malawi. Journal of Infectious Diseases.

[ref-30] Kublin JG, Dzinjalamala FK, Kamwendo DD, Malkin EM, Cortese JF, Martino LM (2002). Molecular markers for failure of sulfadoxine–pyrimethamine and chlorproguanil-dapsone treatment of *Plasmodium falciparum* malaria. International Journal of Infectious Diseases.

[ref-31] Laufer MK, Thesing PC, Eddington ND, Masonga R, Dzinjalamala FK, Takala SL, Taylor TE, Plowe CV (2006). Return of chloroquine antimalarial efficacy in Malawi. New England Journal of Medicine.

[ref-32] Liu DQ, Liu RJ, Ren DX, Gao DQ, Zhang CY, Qui CP, Cai XZ, Ling CF, Song AH, Tang X (1995). Changes in the resistance of *Plasmodium falciparum* to chloroquine in Hainan, China. Bulletin of the World Health Organization.

[ref-33] Malmberg M, Ferreira PE, Tarning J, Ursing J, Ngasala B, Björkman A, Mårtensson A, Gil JP (2013). *Plasmodium falciparum* drug resistance phenotype as assessed by patient antimalarial drug levels and its association with pfmdr1 polymorphisms. Journal of Infectious Diseases.

[ref-34] Mbogo GW, Nankoberanyi S, Tukwasibwe S, Baliraine FN, Nsobya SL, Conrad MD, Rosenthal PJ (2014). Temporal changes in prevalence of molecular markers mediating antimalarial drug resistance in a high malaria transmission setting in Uganda. American Journal of Tropical Medicine and Hygiene.

[ref-35] Mehlotra RK, Fujioka H, Roepe PD, Janneh O, Ursos LM, jacobs Lorena V, McNamara DT, Bockarie MJ, Kazura JW, Kyle DE, Fidock DA, Zimmerman PA (2001). Evolution of a unique *Plasmodium falciparum* chloroquine-resistance phenotype in association with pfcrt polymorphism in Papua New Guinea and South America. Proceedings of the National Academy of Sciences of the United States of America.

[ref-36] Mekonnen SK, Aseffa A, Berhe N, Teklehaymanot T, Clouse RM, Gebru T, Velavan TP (2014). Return of chloroquine-sensitive *Plasmodium falciparum* parasites emergence of chloroquine-resistant *Plasmodium vivax* in Ethiopia. Malaria Journal.

[ref-37] Mita T, Kaneko A, Lum JK, Zungu IL, Tsukahara T, Eto H, Kobayakawa T, Björkman A, Tanabe K (2004). Expansion of wild type allele rather than back mutation in pfcrt explains the recent recovery of chloroquine sensitivity of *Plasmodium falciparum* in Malawi. Molecular and Biochemical Parasitology.

[ref-38] Mohammed A, Ndaro A, Kalinga A, Manjurano A, Mosha JF, Mosha DF, Kavishe RA (2013). Trends in chloroquine resistance Marker, Pfcrt-K76t mutation ten years after chloroquine withdrawal in Tanzania. Malaria Journal.

[ref-39] Moore DV, Lanier JE (1961). Observations on two *Plasmodium falciparum* infections with an abnormal response to chloroquine. American Journal of Tropical Medicine and Hygiene.

[ref-40] Mubjer R, Adeel A, Chance M, Hassan A (2011). Molecular markers of anti-malarial drug resistance in Lahj Governorate, Yemen: baseline data and implications. Malaria Journal.

[ref-41] Mula P, Fernández-Martínez A, De Lucio A, Ramos JM, Reyes F, González V, Benito A, Berzosa P (2011). Detection of high levels of mutations involved in anti-malarial drug resistance in *Plasmodium falciparum* and *Plasmodium vivax* at a rural hospital in southern Ethiopia. Malaria Journal.

[ref-42] Mungthin M, Suwandittakul N, Chaijaroenkul W, Rungsrihirunrat K, Harnyuttanakorn P, Seugorn A, Na Bangchang K (2010). The patterns of mutation and amplification of *Plasmodium falciparum* pfcrt and pfmdr1 genes in Thailand during the year 1988 to 2003. Parasitology Research.

[ref-43] National Information Centre, Yemen (2014). Population growth trends. http://www.yemen-nic.info/sectors/popul.

[ref-44] Nosten F, White NJ (2007). Artemisinin-based combination treatment of falciparum malaria. American Journal of Tropical Medicine and Hygiene.

[ref-45] Nwakanma D, Kheir A, Sowa M, Dunyo S, Jawara M, Pinder M, Milligan P, Walliker D, Babiker HA (2008). High gametocyte complexity and mosquito infectivity of *Plasmodium falciparum* in the Gambia. International Journal for Parasitology.

[ref-46] Reed MB, Saliba KJ, Caruana SR, Kirk K, Cowman AF (2000). Pgh1 modulates sensitivity and resistance to multiple antimalarials in *Plasmodium falciparum*. Nature.

[ref-47] Sisowath C, Ferreira PE, Bustamante LY, Dahlström S, Mårtensson A, Björkman A, Krishna S, Gil JP (2007). The role of pfmdr1 in *Plasmodium falciparum* tolerance to artemether-lumefantrine in Africa. Tropical Medicine & International Health.

[ref-48] Sisowath C, Strömberg J, Mårtensson A, Msellem AM, Obondo C, Björkman A, Gil JP ( 2005). In vivo selection of *Plasmodium falciparum* pfmdr1 86N coding alleles by artemether-lumefantrine (Coartem). Journal of Infectious Diseases.

[ref-49] Thomsen TT, Ishengoma DS, Mmbando BP, Lusingu JP, Vestergaard LS, Theander TG, Lemnge MM, Bygbjerg IC, Alifrangis M (2011). Prevalence of single nucleotide polymorphisms in the *Plasmodium falciparum* multidrug resistance gene (Pfmdr-1) in Korogwe District in Tanzania before and after introduction of artemisinin-based combination therapy. American Journal of Tropical Medicine and Hygiene.

[ref-50] Vieira PP, Urbano Ferreira M, Alecrim MG, Alecrim WD, Da Silva LHP, Sihuincha MM, Zalis MG (2004). Pfcrt polymorphism and the spread of chloroquine resistance in *Plasmodium falciparum*populations across the Amazon Basin. Journal of Infectious Diseases.

[ref-51] Wellems TE, Plowe CV (2001). Chloroquine-resistant malaria. Journal of Infectious Diseases.

[ref-52] White NJ (2004). Antimalarial drug resistance. Journal of Clinical Investigation.

[ref-53] White NJ (2008). Qinghaosu (artemisinin): the price of success. Science.

[ref-54] WHO (2009). Methods for Surveillance of antimalarial drug efficacy.

[ref-55] Wongsrichanalai C, Pickard A, Wernsdorfer W, Meshnick S (2002). Epidemiology of drug-resistant malaria. The Lancet Infectious Diseases.

[ref-56] World Health Organization (2015). World Malaria Report 2015. http://www.who.int/malaria/publications/world-malaria-report-2015/report/en/.

